# A bioethical perspective on the meanings behind a wish to hasten death: a meta-ethnographic review

**DOI:** 10.1186/s12910-024-01018-y

**Published:** 2024-02-27

**Authors:** Paulo J. Borges, Pablo Hernández-Marrero, Sandra Martins Pereira

**Affiliations:** 1 Hospital do Divino Espírito Santo (HDES), Ponta Delgada, São Miguel, Portugal; 2 Instituto de Bioética, Universidade Católica Portuguesa, Porto, Portugal; 3 CEGE: Research Center in Management and Economics – Ethics and Sustainability Research Area, Católica Porto Business School, Universidade Católica Portuguesa, Porto, Portugal; 4 Portuguese Nurses Association for Long-Term and Palliative Care (AECCP), Lisbon, Portugal

**Keywords:** Wish to hasten death, Wish to die, End-of-life care, Ethics, Ethical issues, Principle based ethics, Autonomy, Dignity, Vulnerability, Meta-ethnography

## Abstract

**Background:**

The expressions of a “wish to hasten death” or “wish to die” raise ethical concerns and challenges. These expressions are related to ethical principles intertwined within the field of medical ethics, particularly in end-of-life care. Although some reviews were conducted about this topic, none of them provides an in-depth analysis of the meanings behind the “wish to hasten death/die” based specifically on the ethical principles of autonomy, dignity, and vulnerability. The aim of this review is to understand if and how the meanings behind the “wish to hasten death/die” relate to and are interpreted in light of ethical principles in palliative care.

**Methods:**

We conducted a meta-ethnographic review according to the PRISMA guidelines and aligned with Noblit and Hare’s framework. Searches were performed in three databases, Web of Science, PubMed, CINAHL, with no time restrictions. Original qualitative studies exploring the meanings given by patients, family caregivers and healthcare professionals in any context of palliative and end-of-life care were included. A narrative synthesis was undertaken. PROSPERO registration CRD42023360330.

**Results:**

Out of 893 retrieved articles, 26 were included in the analysis, accounting for the meanings of a total of 2,398 participants. Several factors and meanings associated with the “wish to hasten death” and/or “wish to die” were identified and are mainly of a psychosocial and spiritual nature. The ethical principles of autonomy and dignity were the ones mostly associated with the “wish to hasten death”. Ethical principles were essentially inferred from the content of included articles, although not explicitly stated as bioethical principles.

**Conclusions:**

This meta-ethnographic review shows a reduced number of qualitative studies on the “wish to hasten death” and/or “wish to die” explicitly stating ethical principles. This suggests a lack of bioethical reflection and reasoning in the empirical end-of-life literature and a lack of embedded ethics in clinical practice. There is a need for healthcare professionals to address these topics compassionately and ethically, taking into account the unique perspectives of patients and family members. More qualitative studies on the meanings behind a wish to hasten death, their ethical contours, ethical reasoning, and implications for clinical practice are needed.

**Supplementary Information:**

The online version contains supplementary material available at 10.1186/s12910-024-01018-y.

## Background

The wish to hasten death and associated terms, such as “desire to die”, “wish to die”, or “request to hasten death”, have gained increasing interest in the medical and bioethical literature in the last two decades [1, 2]. In 2016, an international consensus definition was proposed for the “wish to hasten death”, where the terms “wish” and “desire” were used interchangeably [3]. The wish to hasten death was thus defined as a reaction to suffering, where the patient considers that accelerating death is the only way out. This wish is associated to the context of experiencing a life-threatening or life-limiting condition and may be expressed spontaneously or after being queried [3–5]. This concept should be distinguished from the acceptance of impending death or from a wish to die naturally, although preferably soon [3–5].

Evidence shows that the wish to hasten death is associated with various factors, such as physical symptoms, psychological distress (e.g., depression, fears, hopelessness), existential suffering (e.g., tiredness of life, loss of meaning of life), and/or social aspects (e.g., feeling like a burden to others) [1, 3–5]. According to Pestinger et al. [6], three main motivations and meanings underlying a desire to hasten death among patients with advanced disease can be thematized: self-determination, agony, and time [6]. Yet, the expression of a desire to hasten death is not necessarily a synonym for wanting healthcare professionals to practice euthanasia or physician-assisted suicide. Instead, expressing a wish to hasten death is sometimes used as an extreme coping strategy against anticipated agony or as a way to alleviate psychological distress and regain a sense of control [3, 4, 6]. In fact, what many patients who express a wish to hasten death expect from their caregivers is for them to be open so they can share and explore their ideas, emotions and perceptions [6].

The wish to hasten death raises important ethical concerns and is related to ethical principles that are intertwined within the field of medical ethics, particularly in the context of end-of-life care and decision-making. Despite the traditional relevance given to the four main pillars of bioethics and medical ethics (i.e., the ethical principles of autonomy, beneficence, non-maleficence, and justice) [7], other ethical principles, such as dignity, fidelity, and vulnerability, have shown to be paramount in end-of-life care [8, 9]. The latter ethical principles help healthcare professionals and caregivers to better understand and prioritize patients' autonomy, respecting their wishes [7, 8]. Moreover, it is important to recognize the importance of providing compassionate and holistic care, addressing physical, emotional, and spiritual needs of patients and their loved ones. This requires the establishment of a person-centered care plan that provides high-quality end-of-life care, respects patients' autonomy and dignity and offers comfort and support during difficult times [9, 10, 12]. In fact, embracing holistic clinical practices as well as human integrity are crucial when caring for patients with life-threatening and/or life-limiting diseases [7, 9–12].

Autonomy is a foremost ethical principle in healthcare and a core principle in ethical decision-making in palliative and end-of-life care [13–16]. It recognizes that patients have the right to decide about their medical treatment and care, including accepting or refusing medical therapeutics, withdrawing life-sustaining treatment, or requesting medical aid in dying based on their values and goals [10, 14–17]. Healthcare professionals should respect and support patients’ autonomy by providing them with relevant information, ensuring their understanding, and involving them in the decision-making process [14, 16, 18]. Autonomy is a cornerstone in palliative and end-of-life care ethics due to its relevance and implications, namely in terms of anticipating situations and decisions for the future in order to meet patients’ wishes and preferences for care [14, 16].

As a core ethical principle, dignity stands for intrinsic worth and respect for the equality of all individuals as human beings, regardless of their circumstances, age, gender, ethnicity, socioeconomic status, or health condition [19, 20]. Dignity encompasses the idea that everyone deserves to be treated with respect, fairness, and compassion, and raises the need to prize the unique perspectives and experiences of the patients. It ensures that patients’ physical, emotional, and psychological well-being is prioritized and honored [21].

Vulnerability generally refers to a state or condition of being susceptible to harm [22, 23]. It often stems from physical, emotional, and psychological fragility and social factors undermining individuals’ ability to protect themselves or express autonomy [23]. Defining vulnerable persons or populations has proved to be more difficult than initially expected [22–24]. In the context of healthcare, vulnerability can manifest itself in many ways, including individuals’ experiences of illness, injury, disability, and even emotional harm [23]. Social determinants of health, such as poverty, homelessness, or discrimination, can potentiate vulnerability and create additional barriers to accessing healthcare and support [12, 22, 24]. In some cases, the bioethical principles of vulnerability and autonomy seem opposite. However, establishing a dichotomy between these two ethical principles drives to situations where both principles cannot be reconciled [23]. In fact, vulnerability ultimately leads to the establishment of relational autonomy, where vulnerability and autonomy do not stand in opposition to each other [23].

The literature suggests that vulnerability is inherent to situations and not to specific groups of people [23–25]. Also, the location and context of end-of-life care have been associated with vulnerability. For instance, MacArtney et al. [25] point out that patients in palliative care inpatient units conceptualized home as their preferred place of care at the end of their lives, although they perceived a sense of bodily vulnerabilities. In these situations, institutional care was considered as a protection towards their families due to the social, emotional, and relational burdens of dying [24–26]. Patients receiving palliative care often experience extreme vulnerability, which may be perceived at various levels and can be categorized differently [23, 24, 26]. This is a tangible reminder of the fragility of their human condition [23, 26].

Several systematic reviews related to the wish to hasten death and/or desire to die have been performed [4, 19, 20, 27–30]. These reviews focused on different aspects of the wish to hasten death, namely: (i) the feeling of being a burden and its relation to a wish to hasten death [30]; (ii) meaning in life interventions implemented in patients with advanced diseases and their context, mechanisms, and outcomes about a wish to hasten death [5]; (iii) existing instruments for assessing the wish to hasten death [29]; (iv) stakeholders’ perspectives about the wish to hasten death [21]; and (v) reasons and meanings behind a wish to hasten death [4, 19]. Other reviews explored the wish to hasten death in specific medical conditions (e.g., amyotrophic lateral sclerosis) or care settings (e.g., long-term care facilities) [31, 32]. To the best of our knowledge, none of these reviews performed an in-depth appraisal and analysis of the meanings behind the wish to hasten death and/or desire to die in relation to or based on the above-mentioned ethical principles of autonomy, dignity, and vulnerability.

The objective of the study is to understand if and how the meanings behind the “wish to hasten death” and/or “wish to die” relate to and are interpreted in light of the ethical principles of autonomy, dignity, and vulnerability in palliative care.

## Methods

This study consists of a meta-ethnographic review, which is a type of qualitative synthesis that offers a greater description of methods and provides a higher order of interpretation when compared to conventional narrative literature reviews [33–35]. Meta-ethnography is an inductive, interpretative approach based on interpretative qualitative synthesis methods [36]. It is the most utilized qualitative synthesis approach in healthcare research and is particularly suited to developing conceptual models and theories [37, 38]. The seven phases of Noblit and Hare’s [33] framework for the development of a meta-ethnographic review were followed and operationalized (Table 1). PRISMA was used as a reporting guideline [39]. The protocol was registered in PROSPERO (CRD42023360330) [40].

**Table 1:** Systematization of Noblit and Hare’s [33] seven phases of a meta-ethnographic review

**Seven phases of a meta-ethnographic review following Noblit and Hare ** [33]	**Operationalization of the seven phases in this meta-ethnographic review**
1. “Getting started”: choosing the topic focus	The research question resulted from the gap that we identified in the empirical literature about the ethical reasoning associated with the wish to hasten death in palliative care A meta-ethnographic review was considered appropriate and valuable to explore the meanings behind a wish to hasten death in relation to and in light of ethical principles, taking into account the perspectives of patients, family members, and healthcare professionals
2. “Deciding what is relevant to the initial interest”: identifying and selecting study accounts to synthesize	A systematic search was conducted in three databases. The search strategy included MeSH and keywords related to concepts associated with the wish to hasten death and the desire to die. Hand searches complemented the screening of multiple databases Only qualitative studies that fitted the eligibility criteria were included as only qualitative studies could explore meanings. Qualitative data from mixed-methods studies were also included in the analysis
3. “Reading the studies”: repeated reading of study accounts and detailed recording of the concepts, themes, and metaphors	The full reading of the included articles allowed us to extract the raw data and information, and interpret meanings. Concepts, themes, and metaphors were analyzed thoroughly to identify and explore meanings in relation to and in light of ethical principles. This was done through an iterative process with ongoing discussions among all authors
4. “Determining how studies are related”: comparing study accounts by creating and juxtaposing a list of concepts from each study to judge whether the concepts are similar, contradictory, or about different aspects of the topic being researched	For each article, a list of meanings and factors/motivations behind the wish to hasten death was created. The bioethical principles were also identified in the articles, whenever they were mentioned. When not explicitly indicated, an interpretative approach was used to infer ethical principles as appropriate. Accounts from all included studies were critically analyzed and compared to judge whether concepts were similar, contradictory, or complementary
5. “Translating the studies into one another”: systematically comparing or “translating” themes, metaphors, or concepts across and within primary study accounts	Synthesis was conducted based on the type of participant included in each study. Articles were organized according to (i) the type of participants (five subgroups of participants were generated) and (ii) chronological order (from most recent). Please see Table 2. Concepts were compared among included articles and meanings were imbricated with bioethical principles
6. “Synthesizing translations”: When phase 5 results in many translations, these can be compared to see if there are common types of translations or if some translations or concepts can encompass those from other study accounts to reach new interpretations	Results were integrated and evaluated considering the similarities or disparities of concepts. Common themes between studies were summarized and contradictions were evaluated to build a new understanding of the meanings. This allowed us to identify and understand how these bioethical principles are inherent and relate to the main meanings behind the wish to hasten death
7. “Expressing the synthesis”: conveying the findings of the synthesis in a form suitable for the particular audience	The synthesis was reported following PRISMA, as appropriate. A summary of the findings and their interpretation is provided. Please see Fig. 2. Strengths, limitations, and implications for further research, policy, and clinical practice are highlighted

### Eligibility criteria

This review considered empirical and qualitative peer-reviewed articles, written in English, focusing on adults (aged above 18 years old) with a terminal illness and/or at the end of life who experienced and/or verbalized a “wish to hasten death” and/or “desire to die”. Selected studies included patients, family caregivers, and health professionals in any context of palliative and end-of-life care. In case of mixed-methods studies meeting these eligibility criteria, only their qualitative data were included in the analysis.

Studies with adults with mental disorders, pregnant women, and/or individuals with cognitive impairment were excluded. No restrictions were established in terms of the year of publication.

### Search strategy and selection process

The search strategy was discussed and agreed upon among all authors and subsequently applied to three databases, Web of Science, PubMed, and CINAHL. Searches were conducted between January and May 2023. A final search was re-run just before the final analysis and before manuscript submission (October 2023). The search strategy embraced multiple combinations of MeSH and free terms (Additional file 1). Retrieved articles were imported into a reference management software (EndNote 20). Duplicates were removed and references of included studies were also screened for potential additional inclusions.

The first author (P.J.B.) independently selected the potential articles to be included in the review based on the abstracts and titles and proceeded with full-text reading of the selected articles to identify the eligible ones. To minimize bias, one researcher (P.J.B.) screened the first 25% of retrieved articles together with the two other researchers (P.H.M. and S.M.P.). Selected articles were read in full by two researchers independently (P.J.B. and S.M.P.) to identify eligible studies. Doubts were discussed among the three researchers (P.J.B., S.M.P., and P.H.M.) until reaching a consensus.

### Data extraction

A data extraction table was purposively built for this study to extract data from included studies. This form was adapted from previous reviews conducted by members from this research team [41–43] and is based on PICOD [44, 45] as follows: P = Participants:

Patients aged above 18 years old with a terminal illness and/or at the end of life who experienced and/or verbalized a “wish to hasten death” and/or “desire to die”, family caregivers, and health professionals; I = Intervention/phenomenon of Interest: Meanings behind a “wish to hasten death/die” and associated ethical principles; C = Context: Any context of palliative and end-of-life care; O = Outcomes: Meanings and ethical principles; and D = Study Design: Qualitative studies only. Only qualitative data from mixed-methods studies were included in the analysis. Additionally, descriptive data included authors, year of publication, country where the study was conducted, and number of participants in the study (Table 2). One author (P.J.B.) extracted the data in an ongoing, iterative, and dynamic process together with the other two authors (S.M.P. and P.H.M.).

**Table 2:** Characteristics of included articles, using the PICOD [44, 45] framework

**Authors, year, country, and quality score following Hawker et al. ** [65]	**P: Participants**	**I: Intervention(s) / Phenomenon of interest**	**C: Context**	**O: Outcome(s) related to our research objectives**	**D: Study Design**
**Patients**					
Kelly et al. [1] 2002, Australia, 35	72 terminally ill patients with cancer	Factors associated with patients’ wish to hasten death	Hospice unit and a Home Palliative Care Service	Patients with a high wish to hasten death show greater concerns with physical symptoms and psychological suffering, perceive themselves as a burden to others, and experience higher levels of demoralization. They also report less confidence in symptom control, fewer social supports, less satisfaction with life experiences, and fewer religious beliefs when compared with patients who had a moderate or no wish to hasten death. These findings suggest that interventions to improve symptom control, address psychological distress, and enhance social support may help reduce the desire for hastened death among terminally ill cancer patients.	Qualitative analysis using in-depth semi-structured interviews
Coyle and Sculco, [46] 2004, USA, 35	Seven patients with advanced cancer who expressed a desire for hastened death	Meanings and uses of an expressed desire for hastened death in patients with advanced cancer	Urban cancer research center where patients were followed by the Pain and Palliative Care Service	Expression of a desire for hastened death is a complex language and results from the lived experience (physical, psychological, social, spiritual, and existential) of individuals with advanced cancer. Nine distinct meanings were extrapolated from the narratives: “(1) a manifestation of the will to live (paradox); (2) the dying process itself was so difficult that an early death was preferred; (3) the immediate situation was unendurable and required instant action; (4) a hastened death was an option to extract oneself from an unendurable situation; (5) a manifestation of the last control of the dying person; (6) a way of drawing attention to itself as a unique person; (7) a gesture of altruism; (8) an attempt at manipulation of the family to avoid abandonment; and (9) a despairing cry depicting the misery of the current situation.”	A qualitative study with a phenomenological approach using a series of in-depth semi-structured interviews
Johansen et al. [47] 2005, Norway, 35	18 patients with cancer with a short life expectancy	Attitudes towards, and wishes for, euthanasia or physician-assisted suicide in patients with cancer with short life expectancy	Inpatient palliative medicine unit in the Department of Oncology and Radiotherapy of a Hospital in Norway	Wishes for euthanasia or physician-assisted suicide (PAS) are different from requests. Fear of future pain, rather than actual or perceived pain, was the predominant motivation for a possible future wish for euthanasia or physician-assisted suicide among the cancer patients interviewed. Pain was also found to be of major importance for the perceived meaning of life, quality of life, and hopes for the future. There seems to be a clear discrepancy between attitudes, wishes, and requests for euthanasia or PAS and what seems to be the characteristics and nature of wishing for these options among advanced cancer patients. Healthcare professionals should be aware of these discrepancies and take them into account when discussing end-of-life care options with terminally ill cancer patients.	A qualitative study with semi-structured interviews that were analyzed using a grounded theory approach
Eliott and Olver, [48] 2009, Australia, 34	28 terminally ill patients with cancer	Experiences and Perspectives of Dying Cancer Patients Regarding Hope	Palliative Care Units and Hospices in Australia	Hope is a complex and multifaceted concept important to dying cancer patients; different versions of hope can coexist within the same individual. Participants commonly discussed three aspects of hope: (i) hope as interconnectedness: meaning interpersonal connections and relationships with others. This aspect emphasizes the importance of social support and the role that it plays in maintaining hope in the face of a terminal illness (motivation); (ii) hope as realistic optimism: refers to a realistic understanding of the situation while also acknowledging the challenges and limitations of the situation (dynamic process); and (iii) hope as acceptance: expressed through a sense of peace with the inevitability of death, finding meaning and purpose in life, and being able to let go with a sense of dignity and grace. Healthcare professionals should acknowledge and validate patients' hopes while helping them manage unrealistic expectations or fears.	A qualitative approach using in-depth interviews, which were analyzed using a grounded theory approach
Nissim et al. [49] 2009, Canada, 35	27 patients with advanced cancer in ambulatory care	Experience of the desire to hasten death (DHD) in patients with advanced cancer and how it evolves over time	Outpatients from a large cancer center in Toronto, Canada	Three main categories of DHD experiences were identified: (1) contemplation of DHD as a hypothetical exit plan; (2) DHD as an expression of despair triggered by an exacerbation of mental or physical adversity; and (3) DHD as a manifestation of letting go that emerged only in the last weeks of life. These categories were not mutually exclusive, and the same participant described different types of experiences at different moments of the illness.	A longitudinal qualitative approach using in-depth interviews that were analyzed using a grounded theory approach
Vlug et al. [50] 2011, Netherlands, 35	292 ill and impaired participants from a cohort of 5449	Development of a quantitative instrument to measure factors influencing self-perceived dignity in end-of-life care	Adults recruited through the Dutch Association for Voluntary Euthanasia (NVVE) and the Dutch Patients Association (NPV)	Identified factors that affect a patient's self-perceived dignity with an instrument developed to enhance insights into the factors that constitute dignity. The used instrument consists of 26 items and was grouped into four domains: evaluation of self about others (8 aspects), functional status (9 aspects), mental state (4 aspects), and care and situational aspects (5 aspects). Domains I, and IV, mainly contain elements of the categories ‘‘Dignity-conserving repertoire’’ and ‘‘Social dignity inventory’’. Domains II and III largely correspond to the themes and sub-themes of the category ‘‘Illness-related concerns’’ in the model. The instrument developed could help improve dignity-conserving care and research on end-of-life care.	Combination of qualitative and quantitative methods. Open-ended and closed-ended questions about personal dignity were applied resulting in content labels. Only qualitative data were included in this review
Güell et al. [51] 2015, Spain, 34	69 terminally ill patients with cancer, of the 701 patients seen during the study period, succeed in the criteria of making desire-to-die statements (DDSs) to any member of the palliative care unit	Investigate the prevalence of desire-to-die statements among terminally ill cancer patients in an acute palliative care unit and compare the underlying differences between those patients who make desire-to-die comments and those who make desire-for-euthanasia comments	Acute palliative care unit at the Oncology Department of a Spanish Hospital (Barcelona)	The reasons for the desire to die and for euthanasia were mainly nonphysical. Patients who request euthanasia consider themselves less spiritual. Addressing psychological, emotional, and spiritual needs of terminally ill cancer patients receiving palliative care and providing adequate family support is highlighted in the study. Each patient’s life experiences, and individual notions of autonomy and beneficence need to be accounted. Qualitative studies are needed to better understand the great diversity of reasons behind a desire to die.	One-year cross-sectional study with semi-structured interviews. Only qualitative data from this study are included in the analysis
Pestinger et al. [6] 2015, Germany, 35	12 inpatients, mentally not impaired, with an advanced incurable disease, who had made a statement or request to hasten death	Motivations of patients expressing a desire to hasten death (DHD) in a country with prohibitive legislation on euthanasia and physician-assisted suicide	Departments of palliative medicine in three hospitals in Germany	Patients expressing DHD may be motivated by a combination of three main themes: self-determination, agony, and time. The concept of time emerges as a core category. The study also found DHD as an extreme coping strategy to maintain control against anticipated agony. Other relevant topics were identified, such as expectations toward health professionals, attitudes toward death, and secureness related to the end of life. Patients expected health professionals to listen to and respect their experiences, and to provide information about the dying process. Patients also wanted their caregivers to listen to and respect their wish to hasten death but did not expect the caregivers to understand this as an order to hasten their death. Patients wished to be heard and engage in open communication about their DHD.	A qualitative approach with a modified form of Grounded Theory using in-depth interviews
Monforte‐Royo et al. [52] 2018, Spain, 35	193 adult patients, with advanced late-stage disease, cancer that is life-limiting, and/or with a prognosis of 6 to 24 months	Factors that influence end-of-life decision-making for those with advanced cancer, focus on the role of perceived dignity and control in shaping patients' attitudes towards end-of-life care, as well as the impact of functional impairment and symptoms of depression on these attitudes	Oncology unit	The study showed that perceived dignity and control significantly shape patients' attitudes toward end-of-life care, including their desire to hasten death. Specifically, the patients who reported lower levels of perceived dignity and control were more likely to express a wish to hasten death. Therefore, loss of dignity and symptoms of depression are important factors to consider when assessing and intervening with advanced cancer patients, as they are the main antecedents of the wish to hasten death (WTHD).	A mixed quantitative and qualitative approach. Only qualitative data from this study are included in the analysis
Rehmann‐Sutter, [53] 2019, Switzerland, 21	Two patients with cancer	Complex and ethically challenging topic of patients wishing to die because they do not want to be a burden to others	Not specified	Two key meanings are described as a feeling of being a burden to others when it appears as a reason for a wish to die. First, it is an existential kind of suffering insofar as it contains the perception of a plight that is so desperate that it can only be relieved by the end of the patient’s existence. Second, it is an empathic concern that implies caring about the person who bears the burden of care. It is therefore a deeply moral emotion that contains a series of difficulties and challenges, including the adequacy of the representation of the caregiver burden in the patient’s mind and the danger of underestimating the value of one’s life to oneself and others.	Phenomenological approach
**Patients and caregivers, including family members**					
Ohnsorge et al. [54] 2014, Switzerland, 30	30 terminally ill patients with cancer, their caregivers, and relatives	The subjective structure of the WTD statements within three aspects: intentions, motivations, and interactions, on a temporal development approach and social relatedness	Hospice and palliative care wards within the oncology department of a Swiss hospital and outpatients in a palliative care service	Patients were categorized into three groups: (i) Participants who express a WTD with no idea of hastening death; (ii) those who consider a hastened death without undertaking actions that would lead to it; and (iii) those who act towards it WTD statements may differ in their intention through time and can be dynamic and conflicting based on religious and philosophical beliefs, moral and biographic narrative, acute moments of pain, and wishing for quality of life in the terminal phase (autonomy).	Qualitative interviews using Grounded Theory and Interpretive Phenomenological Analysis
Ohnsorge et al. [55] 2014, Switzerland, 34	30 terminally ill patients with cancer, their caregivers, and relatives	Explore the different possible motivations and explanations of patients who express or experience a WTD	Hospice and palliative care ward in the oncology department of a general hospital and an ambulatory palliative care service	Motivations consist of three components: reasons, meanings, and functions Reasons include physical, psychological, social, and spiritual (or existential) aspects. Patients report nine types of meanings: (1) to allow a life-ending process to take its course; (2) to let death put an end to severe suffering; (3) to end a situation that is seen as an unreasonable demand; (4) to spare others from the burden of oneself; (5) to preserve self-determination (autonomy) in the last moments of life; (6) to end a life that is now without value; (7) to move on to another reality; (8) to be an example to others (teaching, dignity); (9) to not have to wait until death arrives. Four types of functions were identified: appeal, vehicle to speak about dying, re-establishing agency, and manipulation.	Semi-structured qualitative interviews were analyzed using a complementary grounded theory and interpretative phenomenological analysis approach
Fornehed et al. [56] 2020, USA, 29	15 patients (> = 40 years old) with a life-limiting illness and 10 family members who were dealing with or had dealt with end-of-life care (EOLC) Ten key informants were family members. Four were family members with a loved one who already died when their interview took place, and six of them had a parent hospitalized	Culturally congruent care that contributes to people’s health and well-being and helps them face disabilities or dying	Homes, hospital rooms, and private conference rooms in Rural Appalachia of Tennessee	Four major themes were deduced from the expressions, patterns, and practices of family decision-making (FDM), accepting death as a part of life, and faith influences at the end of life (EOL): (i) EOL of FDM on the illness trajectory (diagnosis to death) moves at different rates; (ii) Communication encompassing within interdisciplinary team members and families is essential to relieving a burden on someone when making decisions at EOL; (iii) EOL FDM is influenced by a lack of knowledge about the illness trajectory or the need to choose “the lesser of two evils”; (iv) For rural Appalachian’s people, comfort for living while dying means experiencing “a sense of normalcy”.	A qualitative ethno-nursing research method was used to analyze the interviews, guided by Culture Care Theory (CCT)
Young et al. [57] 2021, New Zealand, 34	20 participants (14 people with life-limiting illnesses and life expectancy of less than a year and 6 family members)	Perspectives of patients and relatives on end-of-life practices that hasten death; and ethical distinctions between different practices of assisted dying (AD)	Half of the participants were enrolled in hospice and palliative care in New Zealand. Interviews were carried out in various settings (homes, hospices, and hospitals)	There is limited knowledge about what patients with a life-limiting condition think about end-of-life practices. There is a clear distinction between what health professionals/ethics consider active hastening death and what some patients perceive as such practices. Participants consider end-of-life practices as morally equivalent. Most participants view current palliative care practices, such as pain relief with opioids and symptom management with palliative sedation, as hastening death. Some participants did not agree with the ‘doctrine of double effect’ and saw such practices as ‘slow euthanasia’ and ‘covert euthanasia’. Participants asserted that active and passive practices for ending life were morally equivalent and preferred to choose the time of death over other legal means for death. The study also found that participants' perspectives challenged the interpretation of legal end-of-life practices such as Assisted Dying (AD), which represents an epistemic contest to the foundation of medical knowledge, authority, and ethics.	A qualitative study with interviews
Liu et al. [58] 2021, China, 35	15 adult patients with advanced cancer and 10 family members	Meaning of patient dignity at the end of life in traditional Chinese culture from the perspectives of advanced cancer patients and their family members	Palliative care unit in a hospital in Beijing	Highlight the meaning of dignity for palliative cancer patients and develop a conceptual framework that describes dignity from the perspectives of individuals living with advanced cancer and family members in the traditional Chinese culture. The study identified four categories of dignity: cultural-specific dignity, self-related dignity, family-related dignity, and care- and treatment-related dignity. In traditional Chinese culture, patient dignity at the end of life was relevant to the culture, the individuals, their families, and the care and treatment they received. Patient dignity is supposed to be supported by collaborative efforts from the family and healthcare professionals while considering the patient’s cultural background and personal wishes and values.	A descriptive qualitative design with semi-structured interviews and thematic analysis
**Patients and healthcare professionals**					
Kremeike et al. [59] 2020, Germany, 35	11 adult palliative patients and 149 international expert researchers including clinical practitioners	Development of a clinical approach to face the desire to die in patients receiving palliative care	Department of Palliative Medicine at the University Hospital of Cologne – Germany	Creation of a consensus-based semi-standardized approach for assessing and optimally responding to a desire to die is one of the accomplishments of this study. Eleven patients participated in face-to-face interviews, even if five of them declined to be interviewed for physical or mental deterioration reasons. All but one of the interviewees reported a desire to die either as a wish for hastened death or as acceptance of death without requiring to hasten it. Two interviewees preferred death to deterioration, and a desire for physician-assisted suicide was reported explicitly once. Isolation, feeling as a burden, hopelessness, and fear of pain were also reasons reported for a desire to die Almost all of the interviews appreciate a proactive assessment of the desire to die by health professionals and a trustful health-professional-patient-relationship.	Sequential mixed method design including semi-structured qualitative patient interviews. The agreement consensus was obtained through a two-round Delphi process. Only qualitative data from this study are included in the analysis
Boström et al. [60] 2022, Germany, 34	29 trained health professionals documented and 82 palliative patients	Experiences of trained palliative care providers in open desire die-conversations and identify common themes	Various palliative care settings, including hospices, hospitals, and outpatient services	Desire-to-die conversations are shown to be a positive experience for health professionals, with beneficial aspects such as feeling rewarded when the health professionals see an improvement in their patients, as well as their self-reflection about their role and performance in these conversations. The common main themes that emerged were the importance of an open approach to the desire-to-die conversations, the need for training and support for health professionals in this area, and the challenges associated with addressing suicide in palliative care settings.	Qualitative data using thematic analysis and a concept-driven (deductive) approach
Voltz et al. [61] 2022, Germany, 34	43 health professionals and 85 patients with multiple diagnoses	Conversations between trained health professionals and palliative patients with the development of a desire to die	Ambulatory palliative care settings	Trained communication about the desire to die had effects on several patient-relevant outcomes, including depressiveness, hopelessness, wish to hasten death, death anxiety, patient-health professional relationship, and will to live. In general, the desire-to-die conversations with patients receiving palliative care seem to not lead to harmful effects, however, both risks and benefits were identified. Even if assessed carefully, some trends are pointing toward potentially relieving effects of a desire-to-die conversation in patient depressiveness.	A mixed qualitative and quantitative approach using a three-phase sequential prospective mixed-methods cohort study. Only qualitative data is used in the analysis
**Healthcare professionals**					
Trankle, [62] 2014, Australia, 35	13 physicians who provide end-of-life care	The complexity of influences behind hastened deaths and how physicians reason and experience them	Hospitals and hospices in Australia	Physicians' experiences and understandings of end-of-life care practices are complex and influenced by multiple factors. The study highlights how physicians are affected by politico-religious and cultural influences and medico-legal imperatives with their values and beliefs when making decisions about end-of-life care practices. Physicians regarded the principle of double effect as a simplistic and generalized guideline, that was identified as an ambiguous mechanism to protect physicians who inadvertently or intentionally hastened death. However, all physicians reported it as inadequate as a medico-legal guideline and fundamentally flawed, but sometimes it was their only protective medico-legal option. Physicians are sensitive to the patient ethical principle of autonomy.	A qualitative approach using semi-structured in-depth interviews and thematic analysis
Endacott et al. [63] 2016, England and Israel, 34	55 nurses in intensive care units in Israel and England	Factors perceived to contribute to a “good death” and the quality of end-of-life care in two countries with different legal support and cultural context	Intensive care units in England (3) and Israel (4)	Four common themes that prevent and contribute to a good death and good quality of end-of-life care were identified in both countries. The themes are (i) timing of communication; (ii) accommodating individual behavior; (iii) appropriate care environment, and iv) achieving closure. Nurses have a key role in supporting physicians to communicate with families and patients. Achieve closure is usually more effective for families than for healthcare professionals, who may feel they have not done all. An appropriate care environment allows the patient to die with dignity even if it can be more challenging to the clinical team.	A qualitative study with semi-structured individual and focus group interviews
Galushko et al. [64] 2016, Germany, 31	19 specialized palliative care (physicians, nurses, psycho-social spirituals) professionals	Response of specialized palliative care professionals (SPC) to DHD (desire for hastened death) in daily practice based on their function	Departments of specialized palliative care in four University hospitals	Established the need for better helpful relationships between specialized professionals in palliative care and their patients, through guidelines and training programs to deal with DHD. SPC professionals differ in their relations with the patients and within the team depending on their function. Aspects such as avoidance, understanding and assessing the requests, and evaluating the risk of suicide are some of the aspects that psycho-social spirituals better face without distancing themselves.	Narrative interviews using the documentary method
Hold, [8] 2017, USA, 33	Six experienced hospice nurses	Ethical dilemmas during end-of-life care, resulting in not being able to accurate a good death for patients	Hospice of a metropolitan city in the South-eastern United States	The Institute of Medicine defines a good death as one that is ‘‘free from avoidable distress and suffering for patients, families, and caregivers; in general accord with patients’ and families’ wishes; and reasonably consistent with clinical, cultural and ethical standards’’. To actualize a good death, nurses need to employ an individualized preferences approach to accommodate patients’ wishes and meet their physiological, spiritual, and emotional needs. In this sense, several different ethical quandaries were identified among family members and providers. Nurses use a combination of formal, experiential, and intuitive knowledge to face dilemmas, and three main themes were established: Ethics within Practice, Ethical Knowledge, and Ethical Solutions. Those ethical predicaments were resolved by following either rules (accounting also with their gut feelings) or choosing acts of resistance. This strategy achieved a better understanding of how experienced hospice nurses successfully resolve ethical dilemmas culminating in better deaths for patients.	The qualitative narrative approach is guided by Benner’s “Novice to Expert” as a Theoretical framework
Kremeike et al. [11] 2021, Germany, 35	149 experts in the field of palliative care	Expert opinions surrounding controversies in patients who express the desire to die, to support health professionals in reflecting their attitudes and provide practical conclusions concerning the handling of the desire to die	Scope of palliative care	The panelists’ expert opinions on the topic of the desire to die in palliative care provide insights and practical recommendations into how healthcare professionals should handle these issues. They also concluded that the general public, as well as some vulnerable and high-risk populations (e.g., patients with cancer), despite never having experienced an ad hoc semi-structured clinical interview, highlight the importance of proactive assessment in a persistent desire to hasten death.	Combination of Delphi surveys and qualitative data analysis Only qualitative data is included in the analysis
**Patients, caregivers, and health professionals**					
Ferrand et al. [65] 2012, France, 33	783 participants with requests to hasten death (476 patients, 258 relatives/friends, and 49 nurse staff)	Requests to hasten death (RHD) among patients managed by palliative care teams in a country that widely promotes palliative care	Palliative care organizations	Requests to hasten death are frequent and usually maintained despite appropriate palliative care. These requests are associated with a range of factors, including physical and psychological symptoms (loss of autonomy and dignity, difficulties in feeding, incontinence), social isolation, and existential distress. Most patients who made RHD did not go on to hasten their death. Communication between patients and healthcare professionals was the most important factor in providing appropriate end-of-life care. The study also emphasizes the need for ongoing ethical reflection and dialogue around end-of-life care and requests to hasten death.	Mixed-methods approach, collecting both quantitative and qualitative data Qualitative data was used in the interpretation of the request to hasten death. Only qualitative data is included in the analysis
Ohnsorge et al. [66] 2019, Netherlands, 36	62 palliative patients, their families, and health professionals (total of 248 interviews)	Factors and patterns across dying trajectories that influence palliative patients’ wishes to die (WTD). Insights into how healthcare professionals can better support patients with WTD in palliative care settings	Palliative care facilities	WTD is a complex multifaceted and dynamic phenomenon that is influenced by various factors, including physical symptoms, psychological distress, social support, and existential concerns. The four different typical dying trajectories in focus are neurological diseases, organ failure, frailty due to age, and cancer. The study highlights differences in the experience of WTD depending on the dying trajectory. Patients with neurological diseases report more existential concerns and patients with cancer report more physical symptoms. The feeling of being a burden to others was reported in all patient groups. The study highlights the importance of open communication between patients, families, and healthcare professionals by providing adequate symptom management and psychosocial support about end-of-life care options.	Qualitative semi-structured interviews using phenomenological analysis and grounded theory
Gudat et al. [67] 2019, Sweden, 32	62 patients, their caregivers, and professionals	What terminally ill patients think and intend when experiencing a wish to die (WTD) and feeling like a burden to others	Terminally ill palliative patients	A better understanding of the values and moral sensibilities behind a wish to die is needed among terminally ill patients who feel like a burden to others. Identification of several ethical dilemmas that arise in clinical interactions with these patients, such as balancing respect for autonomy with preventing harm to the patient or others, to provide the best possible quality of life for palliative care patients.	Qualitative prospective approach with interview‐based study and combination of phenomenological analysis and ground theory

### Quality assessment

All the articles included in the analysis were assessed for methodological quality. The quality assessment tool developed by Hawker et al. [68] was used. This is a 9-item tool where the minimum score is 9 and the maximum score is 36 [68]. This tool has been widely used in literature since its nine questions are easily scored to assess the quality of the study and can be transformed into a quantitative scale [41–43, 68–72]. All the included studies were independently assessed by the three reviewers (P.J.B., P.H.M., S.M.P.). Independent scores were averaged and discussed for consensus. Scores are presented in Table 2.

### Data analysis and synthesis

A narrative synthesis was undertaken following Popay et al.’s [69] framework. One author (P.J.B.) performed an initial synthesis, which was then validated and discussed with the other two researchers (P.H.M. and S.M.P.). First, we performed a textual narrative synthesis, systematizing and reporting on study characteristics, context, quality, and findings, using the scope, differences, and similarities among the included articles. Second, we grouped and aggregated data into two main themes aligned with our research objective: (i) meanings behind the wish to hasten death and/or wish to die, and (ii) the relationship between these meanings and the ethical principles of autonomy, dignity, and vulnerability in palliative care.

Findings were organized taking three perspectives into account: (i) patients; (ii) family members; and (iii) healthcare professionals. These perspectives were grouped into five sets taking the different combinations of participants in included studies into account: (a) Patients; (b) Patients and caregivers, including family members; (c) Patients and healthcare professionals; (d) Healthcare professionals; and (e) Patients, caregivers, and health professionals. Table 2 shows the data extracted from the included articles.

## Results

**Fig. 1:**
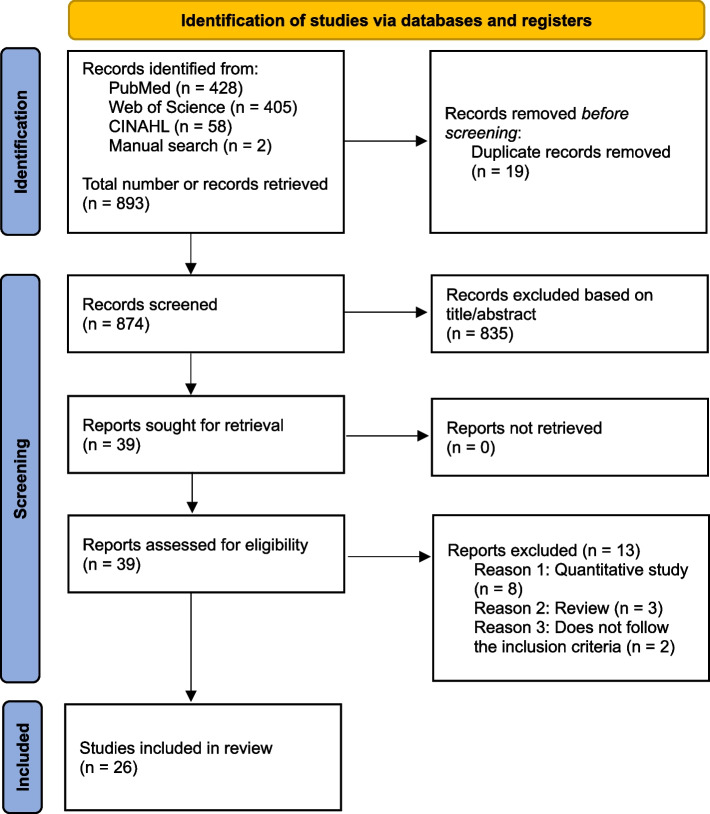
PRISMA flow diagram of the current review

A total number of 893 articles were identified, and 39 full texts remained after screening titles and abstracts. 26 articles were included in the analysis, corresponding to 1,602 patients, 284 caregivers including family members, and 512 healthcare professionals, accounting for a total of 2,398 participants. Figure 1 illustrates the PRISMA flowchart.

### Characteristics of included studies

Twenty-six (*n* = 26) articles were included in the analysis corresponding to twenty years of publications about this topic (2002 to 2022). Most of these studies (*n* = 21) were conducted with patients, although only 10 of them included patients solely [1, 6, 46–53]. Other five articles were performed with patients and caregivers, including family members [54–58]. Five articles included healthcare professionals (physicians, nurses, and psychosocial and spiritual carers) only [8, 11, 61, 63, 64]. Three articles accounted for a combination of the different participants (i.e., patients, caregivers, and health professionals) [56, 65, 67]. Selected studies were conducted in 14 countries, the majority of them (*n* = 8) European. Germany was the country with the highest number of studies (*n* = 6) [6, 11, 59–61, 64].

### Main motivations and factors behind the wish to hasten death

The included studies suggest that the main motivations and factors behind expressing a wish to hasten death are: (i) physical discomfort [1, 47, 50, 54, 55, 59, 65, 67], (ii) unbearable psychological suffering [1, 47, 50, 52, 54, 59, 64, 67], (iii) feeling a burden to others [1, 51–55, 58, 63, 65], (iv) loss of dignity and autonomy [6, 50–54, 62, 65, 67], (v) demoralization and psychological distress [1, 54, 66], (vi) little social support [1, 6, 50, 63, 66], (vii) disappointment with life experiences [47, 54, 55, 63, 67], (viii) tiredness of life [46, 53], (ix) lack of medical options [61], and (x) religious, spiritual and cultural beliefs [49, 54, 56].

### Meanings behind a wish to hasten death

Several meanings emerge associated to the wish to hasten death: (i) early death is preferred over severe and prolonged suffering [1, 49, 55, 59, 64–66], (ii) despair by a miserable exacerbation of mental or physical adversity [6, 52, 53, 55, 58, 65, 66], (iii) fear of future pain [6, 47, 59], (iv) way of escaping from an unendurable and unreasonable situation [51, 53, 55], (v) control the dying process [6, 49, 51, 52, 55–57], (vi) practices of pain relief [59], (vii) dying with dignity [51, 52, 55, 58, 62], (viii) way of drawing attention to him/herself needs [58, 60], (ix) manipulation to avoid abandonment [46], (x) communication strategy to seek information about the dying process [8, 46, 61, 63], (xi) gesture of altruism by ceasing being a burden to others [1, 51–54, 58, 63, 65–67], (xii) hopelessness for the future [11, 47, 52, 59, 61], (xiii) manifestation of the will to live (even if it seems a paradox, it corresponds to the wish to live and being alive while dying) [54, 61], (xiv) detachment, (xv) moving to another reality, and (xvi) being an example to others [55].

### Ethical principles about the wish to hasten death

Ethical principles, such as autonomy, dignity, and vulnerability were not identified in eight of the 26 included articles. Autonomy and dignity were the ethical principles that emerged more frequently, even if most of the time they were inferred from the content of the studies and not explicitly mentioned as bioethical principles [50, 51, 54, 55, 62, 65, 67]. The ethical principles of vulnerability [11] and beneficence [51] as well as the principle of double effect [60] were expressed in only one article each. It was also possible to infer the ethical principle of vulnerability in four other articles [46, 54, 62, 67].

## Discussion

**Fig. 2:**
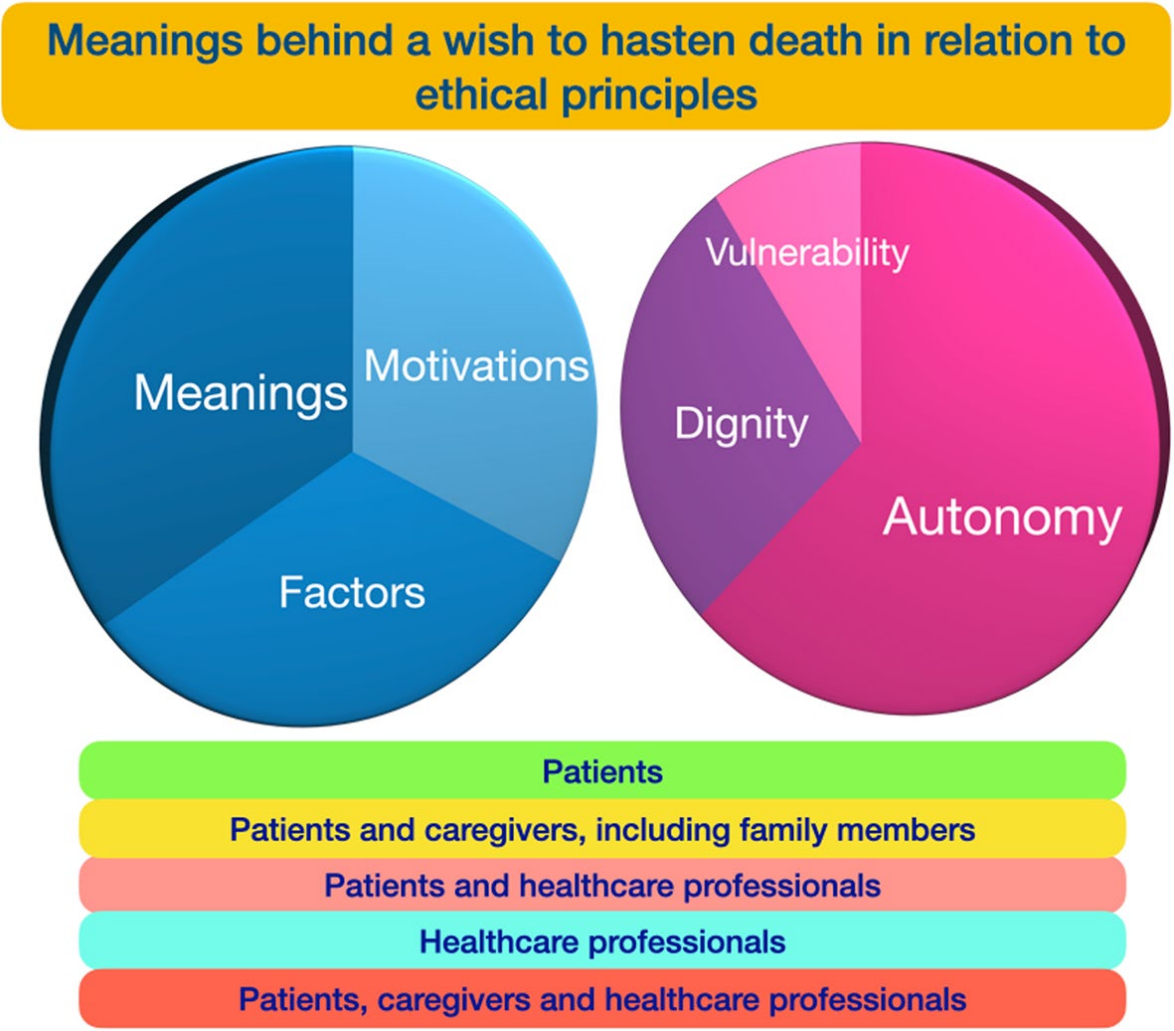
Synthesis of meanings behind a wish to hasten death in relation to the ethical principles of autonomy, dignity and vulnerability

This review shows the wide range of motivations and factors lying behind a wish to hasten death. Most of these are of a psychosocial and spiritual nature. Different meanings were also identified, highlighting the complexity underpinning a wish to hasten death and/or die. While the ethical principles of autonomy, vulnerability and dignity could be inferred from the content of the included studies, only a small number of these studies explicitly revealed the link between an ethical principle and the wish to hasten death and/or die. Autonomy and dignity were the most frequently identified ethical principles. Figure 2 illustrates the synthesis of meanings behind a wish to hasten death in relation to the ethical principles of autonomy, dignity and vulnerability.

### What are the main motivations, factors, and meanings behind a wish to hasten death?

Evidence suggests that there are a series of motivations and factors behind the wish to hasten death. This is not surprising due to the complexity of experiencing a life-threatening and life-limiting condition that increases human vulnerability.

First, feeling a self-burden to others [1, 11, 46, 53, 55, 58, 59, 67], distress [6, 8, 57, 60, 67] and physical symptoms [1, 46, 49, 55, 57, 58, 64, 66, 67] were the main factors underlying a wish to hasten death. Similar results were highlighted in other studies [3–5] showing the relevance of developing care approaches rooted in relational autonomy and not only on individual autonomy [3–5, 70, 73]. Uncontrolled or severe pain, discomfort, and physical deterioration [1, 47, 50, 54, 55, 59, 65, 67] can contribute to a desire for an earlier death as a means to put an end to the patient’s suffering. In some cases, the fear of future pain can be the determinant factor and not the real feeling of pain by itself [47]. Embracing a relational approach (i.e., relational autonomy) [70] and providing truly person-centered care, where fears about the future can be openly discussed and care plans can be defined together with the patient and his/her loved ones, are thus paramount when facing thoughts about of a wish to hasten death/die.

Two key meanings were described by participants in included studies: the feeling of being a burden to oneself and the feeling of being a burden to others [53]. The first is an existential kind of suffering insofar as it contains the perception of a plight where despair can only be relieved by ending a patient’s existence. The second is an empathic concern that implies caring about the person who bears the burden of care. These two feelings can coexist. In reality, feeling a burden to oneself or of being a burden to others can affect perceptions of self-perceived dignity and control. In a sense, these feelings encompass a series of difficulties, challenges, suffering, and despair, which might be exacerbated by the perception of being a burden to others [52, 53]. As a matter of fact, patients tend to internalize these perceptions of burden and feel guilty or responsible for the emotional, financial, or physical strain their illness places on others [25, 30, 53, 55, 58, 67]. Addressing patients’ perceptions is challenging for health professionals. It requires them to embrace a comprehensive approach that includes providing emotional support and helping patients navigate their feelings of burden [1, 61, 63]. Besides, establishing support with empathic communication and counseling [63, 65, 66], involving the patient's support network in the palliative care process to alleviate these perceptions, and exploring alternative ways to find meaning in their lives can bring a sense of reassurance and support.

A second group of factors associated with a wish to hasten death is the presence of unbearable psychological fragility and suffering [1, 47, 58, 66] due to an advanced terminal illness and may result in depression [47, 52], anxiety about the future progression of the disease [47, 56, 61, 66], feelings of hopelessness (despair) [1, 46, 47, 49, 53, 59, 61, 66], and negative emotions such as guilt, shame, or feelings of worthlessness [47, 59]. This can have a powerful impact on patients’ feelings, emotions, and thoughts. In addition, higher levels of demoralization [1, 47, 53, 55], which are commonly associated to a state of hopelessness, despair, and loss of meaning in life [1, 46, 49, 52, 55, 67] psychological distress, depression, anxiety and existential suffering [1, 54, 66], are often present among patients who express a wish to hasten death. All these conditions represent situations of increased vulnerability, which require a careful assessment and ethical sensitivity from healthcare professionals [14, 16] in order to establish and foster a relationship of openness and trust.

Third, patients may also feel that their illness has eroded their dignity and sense of self-worth [50, 52–54, 65, 66] particularly if they are dependent on others for basic care needs resulting in undue distress or loss of bodily functions. Sometimes, patients feel that their illness has stripped them of their independence to make choices or decisions [6, 50, 51, 54, 55, 62, 65, 67]. Quite frequently, these feelings contribute to a desire of regaining control over their own life and death as a way of preserving their remaining dignity. Besides, a sense of weariness or exhaustion caused by the challenges and limitations of their illness, coupled with dissatisfaction stemming from unfulfilled goals, unmet expectations, and despair about the future, can contribute to the desire to hasten death [46, 47, 53–55, 63, 67].

A fourth set of factors is related to social and spiritual dimensions, where the lack of social support or spiritual care together with feelings of isolation can exacerbate loneliness and disconnection from others contributing to the desire to end one’s life [1, 50, 66]. Sometimes, caregivers may feel overwhelmed, resentful, or unable to meet the patient’s needs. This can result in inadequate support and abandonment, which can be increased if healthcare professionals also consider patients as a burden. In these complex circumstances, feelings of worthlessness, hopelessness, and a loss of agency in patients can emerge [54]. By recognizing and addressing their own biases and perceptions, healthcare professionals can create a supportive and compassionate environment that promotes honest open communication and shared decision-making [6, 54, 66].

Existential and spiritual beliefs about the meaning of life, the afterlife, or the concept of a "good death" can also influence expressions of a wish to hasten death. While patients may view death as a natural part of the human experience and wish to embrace it on their own terms, evidence suggests that patients with a high wish to hasten death reported less attachment to their religious, moral, and cultural beliefs [4, 5, 27, 54]. This suggests a shift in values or societal attitudes toward illness and aging that may influence the desire to hasten death [49, 54, 56].

Close to these social and spiritual factors, communication was also identified as a relevant factor associated with a wish to hasten death. In fact, some patients reported disappointment related to the lack of communication with their healthcare professionals. This suggests that patients value competent clinicians who have good communication competencies and offer adequate emotional support [6, 8, 46, 52, 61–66]. Open communication among patients, families and professionals is a core element of palliative and end-of-life care [6, 14, 16, 59, 63, 64, 66]. The desire-for-die conversations showed to be a proactively positive experience for healthcare professionals [60].

A final set of factors relies on clinicians’ political, cultural and ethical values and actions. For instance, the literature points out towards the influence of medico-legal imperatives coupled with clinicians’ values and religious, spiritual and philosophical beliefs when making decisions about end-of-life care practices, such as when a patient expresses a wish to hasten death [62, 63, 67, 74, 75]. It is worth mentioning that there seems to be a discordance between what healthcare professionals and ethicists consider to be the active hastening of death and what some patients and families perceive as such practices [6, 57].

In sum, while the motivations and factors behind a wish to hasten death may not be exhaustive, its meanings can be influenced by a combination of several complex factors. A wish to hasten death results from a unique multifaceted and complex personal experience. Understanding these underlying meanings can help healthcare professionals provide appropriate support and interventions to address patients’ wishes, desires and actual care needs.

### How do the ethical principles of autonomy, dignity and vulnerability relate to and help to interpret meanings behind a wish to hasten death in palliative care?

As aforementioned, only a small number of studies explicitly revealed the link between an ethical principle, mostly autonomy, and the wish to hasten death. Moreover, the ethical principles of autonomy, dignity, and vulnerability could be inferred, and thus interpreted, from the content of most of the included studies. As a matter of fact, even if bioethical principles are imbricated when exploring the meanings behind a wish to hasten death, there seems to be a lack of clarity in articulating these principles within an ethical reasoning and debate framework in the empirical literature.

#### The ethical principle of autonomy in relation to a wish to hasten death

First, the ethical principle of *autonomy*, which is defined as the ability and right of individuals to make fully informed decisions about their medical treatment and care [1, 76], is closely related to a patient’s preference for a hastened death [64]. Indeed, evidence suggests that patients who express a wish to hasten death may be seeking to maintain control over their lives and their deaths [6, 47], wishing to decide how and when they would die [51]. Autonomy is also a cornerstone of person-centered care, which is particularly visible in palliative care [13, 16].

Patients who feel like a burden to others may experience a conflict between their desire to die and their desire to respect the autonomy or independence of their loved ones. While this meaning suggests individual autonomy as a core principle behind a wish to hasten death, it also highlights the relational dimension of autonomy. Relational autonomy aims to maintain the essential aspect of autonomy, namely control over one’s life, while, at the same time, incorporating insights of a socially embedded perception and reality [12, 70].

In this sense, the expression of desire for hastened death can be understood also as a communication tool for patients [46]. On the one hand, it allows patients to express their innermost thoughts and feelings and can be a way to convey the depth of their pain, despair, or desire for relief. On the other hand, the desire to hasten death can be a means for patients to draw attention to themselves and their needs within the healthcare system. By expressing a desire to hasten death, patients may hope to be seen, listened to, and understood in depth about their experiences, losses, and suffering.

In fact, respecting the right to make decisions about their care, including their expressed desire to hasten death, emphasizes patients’ autonomy by recognizing their capacity to make choices. This relates to the foundational concept of this bioethical principle [76]. It is thus paramount to reconceptualize and operationalize the ethical principle of autonomy, including relational autonomy, into end-of-life care practices, particularly those associated with a wish to hasten death.

According to the bioethical literature, true autonomy requires individuals’ choices to be free from undue influence, coercion, or external control. This condition is disputable in situations where a patient is experiencing unbearable pain or suffering. Our review suggests that some patients request anticipated death as a way to avoid prolonged suffering and to manage the fear of future pain and of perceived lack of quality of life [47, 49]. Providing adequate pain relief, palliative care, and psychological support to alleviate unnecessary suffering and improve the quality of life is inherently linked to ensuring a true expression of autonomy and is, additionally, consistent with the principles of beneficence and non-maleficence [76].

#### The ethical principle of dignity in relation to a wish to hasten death

Second, the ethical principle of *dignity* was also identified in this study as inherent to a wish to hasten death when perceived, by the patient, as being compromised. Indeed, patients may feel that their illness has eroded their dignity, particularly if they are dependent on others for basic care or if their condition causes embarrassment or loss of bodily functions [1, 77]. Witnessing their own body in decline and facing others’ reactions to their body decline is a matter of great concern for terminally ill patients [77]. The negative emotions related to having an altered physical appearance have a detrimental impact on the self-identity of these patients [77], affecting their sense of personal dignity. In addition, the perceived lack of quality of life may compromise patients’ dignity both due to their illness and to the limitations of available treatments [49].

From an ethical point of view, the principle of dignity can be framed around three fundamental facets: (i) the uniqueness value inherent in each person, conceived as an end in itself and not as a means (the Kantian concept of a categorical imperative [57]), (ii) persons’ perception of their worth, and (iii) persons’ sense of preservation of their self. Patients’ self-perceived dignity is thus a central goal in palliative care as patients may feel that their illness or condition has robbed their dignity, and they are no longer able to live a life that is meaningful or fulfilling. This can lead to feelings of hopelessness and despair and may contribute to a desire to end their life [50, 52, 53]. It is therefore not surprising that the meaning of dignity at the end-of-life has been mostly explored around the concept of dying with dignity and freedom from pain and suffering both as an ethical imperative and a human right, focusing on how to provide holistic care needs at this vulnerable time in a person’s life [78]. A dignified death includes respecting patients' values, beliefs, unique worldviews, social traditions, and cultural background [56, 58]. Life as an intrinsic good, which is inherent to the ethical principle of dignity, and a peaceful death are also important values [8, 79]. Interventions have been described in the literature to reduce psychosocial and existential distress, and promote dignity, meaning, hope, and peace of mind in patients at the end-of-life [78, 80, 81]. Hence, recognizing dignity while embracing a person-centered care approach can help professionals to identify and consequently address feelings and perceptions of compromised dignity.

#### The ethical principle of vulnerability in relation to a wish to hasten death

Finally, the ethical principle of *vulnerability* was the less referred one in the reviewed literature about the meanings behind a wish to hasten death, even if it was possible to infer its connection with the latter. In fact, terminally ill patients are often in a vulnerable state due to their physical, emotional, psychological, spiritual, and social care needs, as well as their dependence on healthcare providers and caregivers. As an ethical principle, vulnerability is a complex and multifaceted concept that can be influenced by both internal or individual factors, such as age, health status, or cognitive ability, and external or contextual factors, such as social, economic, or political conditions. Vulnerability can be defined as a substantial incapacity to protect one's interests and is a fundamental aspect of human existence, relevant to healthcare practices since vulnerable people need special protection [22–24, 82–84]. Vulnerable individuals or groups are those who are more likely to have their interests unjustly considered [84]. As an ethical principle it therefore emphasizes the need to protect and respect the rights and welfare of vulnerable persons or groups. Moreover, it requires them to be treated as autonomous agents in their decisions and to ensure that they are not subjected to undue risks, harm, or exploitation. Being dependent on others for their care and support, experiencing significant pain, and discomfort, and struggling with feelings of guilt, shame, worthlessness, or other symptoms related to their illness or condition can further increase patients’ sense of vulnerability [11, 49, 53, 55, 57, 58]. Therefore, in what refers to the expression of a wish to hasten death, vulnerability highlights the need to protect and support these individuals, ensuring that their rights, autonomy, dignity, and well-being are respected.

### How can ethical principles help healthcare professionals understand the meanings behind a wish to hasten death?

In this review, healthcare professionals’ meanings and reactions towards a patient’s wish to hasten death were also identified and explored both from their perspectives and from the patients’ ones [6, 57, 62–64]. Findings suggest that patients expressing the desire to hasten death do not necessarily expect healthcare professionals to actively hasten their death, but rather seek understanding, support, and information about the dying process and maintain control over their lives (autonomy) [5, 6, 11]. Frequently, patients’ wish to hasten death evolves and their main preferences change over time, depending on the situation, sometimes showing dramatic changes from an explicit desire to hasten death to a newly experienced wish to live [54]. Establishing respectful communication and proactively addressing desires to die are a way to open up intimate and meaningful conversations, being thus a relevant intervention in palliative care [6, 11]. This calls for professionals to be attentive and ethically sensitive towards patients’ wishes (autonomy) and needs (vulnerability), while simultaneously reflecting on the ethical and legal questions inherent to their duty to preserve, promote and protect life [5, 6, 11, 14, 16, 49, 62, 79, 83, 84].

The importance of healthcare professionals respecting patients’ autonomy is highlighted in several studies [1, 48, 54, 59]. This can be achieved by providing patients with relevant, accurate, and clear information, ensuring their understanding, and involving them in decision-making [1, 48, 54, 59]. Indeed, adequate communication and empathy are crucial to patients with a desire to die as they empower their autonomy and address their unique needs (dignity) and preferences [6, 8, 52]. Discussing end-of-life options can reduce patients and families’ anxiety and uncertainty (which increase their vulnerability), helping them to feel empowered in making decisions (autonomy) and promoting a sense of dignity [47, 54, 56, 61, 66, 85, 86]. In fact, evidence suggests that patients and families often have ethical concerns, which they would like to openly discuss with their attending clinicians, expecting them to be receptive and attentive to having such conversations [87].

To this end, healthcare professionals can help to preserve and enhance patients' sense of self-worth and self-respect by providing personalized care plans, which may even minimize the risk of a perceived loss of dignity. This is aligned with professionals’ aspiration to minimize patients’ suffering and promote their autonomy. Nevertheless, the role of these professionals in end-of-life situations must be carefully observed since physicians' experiences with ethico-clinical decisions to hasten death are affected by a multitude of factors. Political, religious, and socio-cultural beliefs, medico-legal imperatives, personal values and beliefs, and interpersonal and intrapsychic aspects play a major role in end-of-life decision-making [14, 16, 62, 86]. Healthcare professionals sought ethical solutions that prioritized patients' well-being and autonomy, while also navigating through the complex ethical dilemmas that arise in end-of-life care [8, 14]. It is important to acknowledge the duality that the wish to die and the wish to live may rise simultaneously in the same person [11]. Within this duality, professionals may play an important role in developing trustful relationships with patients and families, allowing them to truly respect patients' autonomy and supporting them in making informed decisions consistent with their values, preferences, and concerns.

Finally, holistic and compassionate patient-centered care should be provided while taking the unique needs and perspectives of each individual into account. Divergent opinions can occur among different stakeholders on how to best deal with the desire to hasten death/die. This reflects the tension that co-exists within the triadic professional-patient-family relationship. First, by acknowledging divergent experiences (e.g., wanting to live versus having the desire to die). Second, by taking into account conflicting values (e.g., the protection of the sanctity of life versus respect for the patient’s autonomy).

### What does this review add?

To the best of our knowledge, this is the first meta-ethnographic review conducting an in-depth appraisal and analysis on “if” and “how” the meanings behind a “wish to hasten death/die” relate to and are interpreted in light of the ethical principles of autonomy, dignity, and vulnerability in palliative care. According to our findings, a wish to hasten death is rooted in various motivations, factors, and meanings, which are highly subjective and inherently personal. The same individual (patient, family, healthcare professional) may experience dual thoughts and feelings about this concept, which can be related to several ethical principles. Surprisingly, while an expressed wish for hastened death is commonly anchored in the ethical principle of autonomy, the empirical literature is not framed or explicitly built around core ethical principles, such as autonomy, dignity, and vulnerability.

### Strengths and limitations

The major strength of this review is that it fills a gap in research by interpreting existing evidence about the meanings evoked by participants (patients, family members, and healthcare professionals) about the “wish to hasten death” in light of the ethical principles of autonomy, dignity, and vulnerability. This tridimensionality of included participants in the original studies brings more in-depth, breadth and complementarity to the understanding of this phenomenon by grouping and combining five sets of participants: Patients; patients and caregivers, including family members; patients and healthcare professionals; healthcare professionals; and patients, caregivers, and health professionals.

Nevertheless, it is important to take some limitations into account. First, most of the articles included in our analysis focused on inpatients in hospice care, with advanced and terminal illnesses. On the one hand, this may reflect the inclusion of studies conducted with vulnerable patients. On the other hand, this also corresponds to a very specific context of care where specialized palliative care is commonly provided. Second, none of the articles focused on family caregivers only. Finally, most of the included articles were conducted in European countries that are undergoing legislative processes about different end-of-life practices (e.g., advance directives, advance care planning, euthanasia, assisted suicide).

## Conclusions

This meta-ethnographic review shows a reduced number of qualitative studies on the “wish to hasten death” and/or “wish to die” explicitly stating ethical principles. This suggests a lack of bioethical reflection and reasoning in the empirical end-of-life literature and a lack of embedded ethics in clinical practice. This review also highlights that the wish to hasten death has multiple meanings and may be affected by several factors, such as physical symptoms, psychological/existential suffering, loss of dignity, and feeling a burden to others. While often not mentioned explicitly or with enough in-depth, these meanings relate to the ethical principles of vulnerability, dignity, and autonomy. That is why healthcare professionals should address these topics compassionately and ethically, taking into account the unique perspectives of patients and family members as well as their own internal tensions. Finally, this meta-ethnographic review reinforces the need for more qualitative studies on the meanings behind a wish to hasten death, their ethical contours, ethical reasoning, and implications for clinical practice.

### Supplementary Information

**Additional file 1.** Search strategy.

**Additional file 2.** PRISMA_2020_checklist.

## Data Availability

All data generated or analyzed during this review are included in this published article and its supplementary material.
