# Supplementary material for: A bioethical perspective on the meanings behind a wish to hasten death: a meta-ethnographic review
Source: BMC Med Ethics. 2024 Feb 27;25:23. doi: 10.1186/s12910-024-01018-y (PMC10898028; doi:10.1186/s12910-024-01018-y)
Supplement: Supplementary file 1 — Additional file 1. Search strategy. [file 12910_2024_1018_MOESM1_ESM.docx]

**Supplementary file 1. Search strategies**

| **Search strategy 1** |
| --- |
| **PubMed:**  (((((((([(Wish to hasten death[MeSH Terms]) OR (Desire for hastened death[MeSH Terms])) OR (Desire for death[MeSH Terms])) OR (Wish to die[MeSH Terms])) OR (Hasten death[MeSH Terms])) OR (End-of-life decisions[MeSH Terms])) OR (Desire for early death[MeSH Terms])) OR (Decisions of end of life[MeSH Terms])))))))) AND ((((([(Palliative care[MeSH Terms])) OR (Advanced illness[MeSH Terms])) OR (Terminally ill[MeSH Terms])) OR (Life-threatening condition[MeSH Terms])) OR (Advanced disease[MeSH Terms])])))))  **Web of Science and CINAHL:**  (Wish to hasten death OR Desire for hastened death OR Desire for death OR Wish to die OR Hasten death OR End-of-life decisions OR Desire for early death OR Decisions of end of life) AND (Palliative care OR Advanced illness OR Terminally ill OR Life-threatening condition OR Advanced disease) [free terms on Title/Abstract] |
| **Search strategy 2**  **PubMed:**  Desire to die [free terms on Title/Abstract] |
